# Short Report: The Variants in *CHEK2* in Metastatic Uveal Melanoma

**DOI:** 10.3390/jcm14082815

**Published:** 2025-04-18

**Authors:** Mizue Terai, Rino Seedor, Usman Ashraf, Gretchen Hubbard, Sergei Koshkin, Marlana Orloff, Takami Sato

**Affiliations:** 1 Department of Medical Oncology, Sidney Kimmel Comprehensive Cancer Center at Thomas Jefferson University, Philadelphia, PA 19107, USA; rino.seeder@jefferson.edu (R.S.); sergei.koshkin@jefferson.edu (S.K.); marlana.orloff@jefferson.edu (M.O.); takami.sato@jefferson.edu (T.S.); 2 Caris Life Sciences, Irving, TX 75039, USA; uashraf@carisls.com (U.A.); ghubbard@carisls.com (G.H.)

**Keywords:** uveal melanoma, ocular melanoma, CHEK2, GNA11, GNAQ, BAP1, ATM, PARP, ATR, DNA repair

## Abstract

**Background:** Uveal melanoma (UM) is a rare subtype of melanoma with distinct clinical and molecular features compared to other melanoma subtypes. UM tumors are frequently detected with mutations in *GNA11*, *GNAQ*, *EIF1AX*, *BAP1*, and *SF3B1* instead of the typical mutations associated with cutaneous melanoma. Although hereditary UM is rare, germline *BAP1* loss predisposes patients to UM and various other cancers. The *CHEK2* (Checkpoint kinase 2) gene that encodes the protein CHK2, a serine-threonine kinase, is a cell cycle checkpoint regulator that acts as a tumor suppressor. CHK2 is involved in DNA repair, cell cycle arrest, or apoptosis in response to DNA damage. *CHEK2* mutations have been linked to various cancers. While there is no strong evidence that *CHEK2* mutations increase the risk of melanoma, two cases of germline *CHEK2* mutations in UM patients have been reported. However, the incidence of *CHEK2* variants in metastatic UM (MUM) has not been investigated. Thus, we conducted a retrospective analysis of patients with MUM and *CHEK2* variants to understand this link better. **Methods:** We collected MUM cases from 2016 to 2024 from institutional databases. Tissues underwent analyses of molecular and genomic features, including tumor mutational burden, and were performed by a Clinically Certified Laboratory. Next-generation sequencing and variant calling were conducted to identify *CHEK2* variants. **Results:** In this study, we reported ten patients with *CHEK2* variants among 740 metastatic UM patients (1.4%) and four primary UM patients with *CHEK2* germline mutations. **Conclusions:** Although rare, UM patients with an abnormal ATM–CHEK2 axis might receive clinical benefits from medications that target DNA repair mechanisms.

## 1. Introduction

Uveal melanoma (UM), the most common primary ocular malignancy in adults, originates from melanocytes derived from neural crest cells and later inhabits the choroid, ciliary body, and iris. The cause of UM remains unknown. UM is particularly prevalent in Caucasians, with increased incidence observed in older age groups. The median age at diagnosis of primary UM ranges from 58 to 61 years [1,2,3]. Notably, UM typically has a lower mutational burden compared to cutaneous melanoma [4]. The most frequent genetic mutations are in guanine nucleotide-binding protein alpha 11 (*GNA11*) and guanine nucleotide-binding protein q polypeptide (*GNAQ*), which are somatic in tumor cells. Both *GNA11* and *GNAQ* genes encode proteins involved in the Gα signaling pathway, and their expression is mutually exclusive in most tumors.

Cancer prognosis can be determined based on the mutation status of additional genes like BRCA1-associated protein-1 (*BAP1*), Splicing Factor 3B Subunit 1 (*SF3B1*), and eukaryotic translation initiation factor 1A x-linked (*EIF1AX*). For instance, patients with a *BAP1* mutation have a high risk of metastasis, while patients with an *EIF1AX* mutation have a low risk [5,6]. Germline mutations or variants can be passed from generation to generation (inheritance) and can cause a specific phenotype or disease. A small group of UM patients has been found to harbor genes of familial predisposition. *BAP1* has been identified as one of these predisposition genes, associated with a high susceptibility to various malignancies such as UM, malignant mesothelioma, cutaneous melanoma, renal cell carcinoma, meningioma, and other cancers [7,8,9]. Other germline mutations in UM have been found in the *BRCA2*, *TP53*, and *MBD4* genes, but these mutations are rare, and their roles need further investigation [10,11,12].

**Figure 1:**
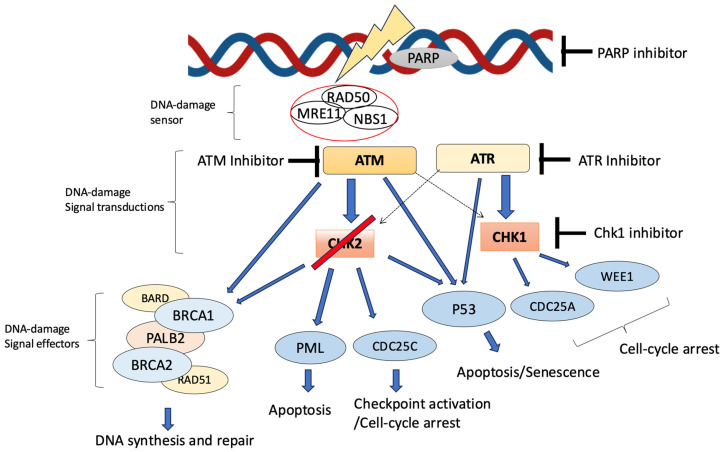
Schematic model of DNA damage response (DDR) signaling pathway and DDR inhibitors used in clinical settings. In response to DNA damage, the PARP protein increases and rearranges DNA damage. In conjunction with the MRN complex, comprising MRE11, RAD50, and NBS1, ATM phosphorylates both CHK2 and TP53. ATM-dependent CHK2 phosphorylation promotes the subsequent CHK2-dependent phosphorylation of numerous downstream substrates such as p53, CDC25C, PML, and a complex of DNA-damage signal effectors, BRCA1/2, PALB2, RAD51, and BARD. CHK2 regulates several cellular processes, such as cell cycle arrest, apoptosis, and DNA synthesis and repair. ATR and CHK1 are activated by junctions between single-stranded DNA (ssDNA) and double-stranded DNA (dsDNA). These genes constitute the main signal for DNA repair mechanisms. CHK1 may also be activated by ATM. These genes collectively regulate cell cycle checkpoint response and apoptosis.

The *CHEK2* gene is located on chromosome 22q12.1 and encodes the CHK2 protein, which is involved in responding to DNA double-strand breaks [13]. Human CHK2 is a serine-threonine kinase and consists of 543 amino acids with three distinct functional domains: an N-terminal SQ/TQ cluster domain (SCD), a forkhead-associated domain (FHA), and a C-terminal Ser/Thr kinase domain [14]. CHK2 is activated by signals from ataxia-telangiectasia mutated kinase (ATM) (Figure 1), which is phosphorylated in response to double-stranded DNA breaks, DNA alkylation, or replicative stress. The ATM–CHEK2-p53 axis has been documented as a backbone for DNA damage response (DDR) and hypothesized as a barrier against cancer initiation [15]. CHK2 contributes to the phosphorylation of downstream target CDC25 to help regulate cell cycle checkpoints and apoptosis [16]. Thus, *CHEK2* mutations severely impair DNA damage repair and cell cycle control. These mutations may impact tumor mutational burden (TMB) and microsatellite instability (MSI), both of which are critical indicators of genomic instability and potential predictors of response to immunotherapies [17]. Pathogenic *CHEK2* variants are associated with an increased risk of several malignancies, including breast, colorectal, prostate, kidney, bladder, and thyroid cancers, depending on whether the mutation is a frameshift mutation or a missense substitution [18]. The two most frequent *CHEK2* variants are missense p.I157T (c.470T > C) and truncating c.1100delC (p.T367Mfs*) [16].

To our knowledge, two cases of germline *CHEK2* and *PALB2* mutations in patients in UM were reported previously [19]. However, the actual incidence of *CHEK2* mutation and variants in UM has not been investigated. Identifying a link between *CHEK2* mutations and MUM may have important implications for both early diagnosis and targeted treatment, potentially improving patient outcomes. In this short report, we retrospectively analyzed and reported 10 metastatic uveal melanoma (MUM) patients with *CHEK2* variants among 740 MUM cases (1.4%) in our MUM database and Caris database.

## 2. Materials and Methods

### 2.1. Participants and Ethical Standards

This study was a retrospective analysis of MUM patients with *CHEK2* variants. The Institutional Review Board (IRB) approved this investigation (Jefferson iRISID-2024-3144). The data were obtained from a clinical laboratory system at Thomas Jefferson University and the database of MUM patients from Caris Life Sciences (Phoenix, AZ, USA).

### 2.2. Patient Specimens and DNA Sequencing

We collected data on MUM cases between 2016 and 2024 from the databases of Thomas Jefferson University and sequenced database Caris Life Sciences (Phoenix, AZ, USA). MUM tissue specimens were retrieved from paraffin-embedded archival core biopsy or surgically removed specimens for mutation analysis. Analyses of molecular and genomic features, including tumor mutational burden, were performed by a CLIA/CAP-certified lab (Caris Life Sciences). Next-generation sequencing (NGS; NextSeq or NovaSeq 6000, Illumina, Inc, San Diego, CA, USA) was performed on genomic DNA using a 592-gene panel and whole-exome sequencing (WES), with over 700 genes analyzed at high coverage and read depth. Genetic variant calling was conducted by board-certified molecular geneticists, as previously described [20]. TMB and MSI were calculated and called as previously described [21,22]. We also collected data on UM patients without systemic recurrence who underwent germline mutation testing.

## 3. Results

### 3.1. UM Patients with Metastasis

First, we identified three cases with variants in *CHEK2* from our institutional database of 162 MUM patients (1.9%) with available NGS and WES of metastatic UM specimens. The following is a summary of three cases from the database at Thomas Jefferson University. Table 1 and Supplementary Table S1 show the summary of key DNA mutations.

#### 3.1.1. Case 1

A 61-year-old male presented with metastasis in the liver. He was diagnosed with choroidal melanoma at the age of 59 and treated with radioactive plaque. A biopsy of the liver tumor was performed three months after the initial diagnosis of primary UM, confirming he had MUM. The tumor had mutations in *GNA11* (p.Q209P) and *BAP1* (p.S583fs). In addition, a likely pathogenic missense variant in *CHEK2* (p.I157T) and a *BRCA2* (c.475 + 1G > T) mutation were found. The total mutational load in the metastatic tissue was 11 mut/Mb and MSI was deemed stable. His familial history showed that his father had leukemia. The patient was treated with immunoembolization with granulocyte-macrophage colony-stimulating factor (GM-CSF) and interleukin-2. Upon disease progression, he was treated with percutaneous hepatic perfusion with HEPZATO KIT™, SIR-Spheres^®^ Y-90 resin microspheres, and chemoembolization with Carmustine (BCNU) to the liver [23,24,25]. The patient passed away 1 year and 10 months after the diagnosis of metastasis.

#### 3.1.2. Case 2

A 67-year-old male presented with MUM in the liver. He was found to have a choroidal nevus in the right eye in his 40s. At the age of 53, he was diagnosed with choroidal melanoma and subsequently treated with radioactive plaque. A biopsy of the liver tumor performed 14 years after his initial diagnosis of primary UM confirmed he had MUM. The tumor had mutations in *GNA11* (p.Q209L) and *BAP1* (c.68-13_82del28). In addition, an *MITF* (c.952G > A/p.E318K) mutation, a *CHEK2* (c.904G > A/p.E302K) variant of uncertain significance (VUS), a *BRCA1* (p.D695Y) mutation, and a deletion of *PALB2* were found. The total mutational load in the tumor tissue was 4 mut/Mb and MSI was stable. The patient’s familial history showed that his mother had stomach cancer, his brother had Hodgkin’s lymphoma, his maternal grandmother had pancreatic cancer, and his maternal grandfather had colon cancer. The patient was treated with chemoembolization with BCNU and was briefly on a clinical trial with FHD-286 [NCT04879071]. The patient passed away 1 year and 4 months after the diagnosis of metastasis.

#### 3.1.3. Case 3

A 57-year-old male presented with MUM in the liver. He was found to have choroidal melanoma in the left eye at the age of 55 and subsequently treated with radioactive plaque. A biopsy of the liver tumor performed 1 year and 4 months after his initial diagnosis of primary UM confirmed the diagnosis of MUM. The tumor had mutations in *GNAQ* (p.Q209P) and *BAP1* (p.P510fs). In addition, we also found pathogenic mutations in *CHEK2* (p.S428F) and *RAD51C* (p.Y216fs). The total mutational load in the MUM tissue was 1 mut/Mb and MSI was stable. The patient’s familial history showed that his father died of glioblastoma. His mother had skin melanoma and died of lung cancer. His sister also had skin melanoma. The patient was enrolled in a clinical trial with a PKC inhibitor [NCT05987332] and is alive.

### 3.2. CHEK2 Variants in the Caris Database

Subsequently, we identified *CHEK2* variants in seven additional MUM cases from the Caris Life Sciences tumor database between 2016 and 2023. We confirmed that these variants were derived from melanoma cells in MUM tissue specimens. *CHEK2* variants were found in seven specimens from 578 cases (1.2%). The mutations of Gαq family members, *BAP1*, *SF3B1*, and *EIF1AX*, were present in the tumor specimens of these patients except for one case (Case 7 in Table 1 and Supplementary Table S1). This patient had a *PLCB4* (p.G517E) mutation. *ATM* somatic or germline mutations were not identified in these patients.

Combining our database with the Caris database, we identified 10 patients with *CHEK2* variants among 740 MUM patients (1.4%).

**Table 1:** The summary of key DNA variants or deletions.

ID	1	2	3	4	5	6	7	8	9	10	11	12	13	14
Group														
CHEK2														
BRCA2														
BRCA1														
RAD51C														
PALB2														
GNA11														
GNAQ														
PLCB4														
BAP1														
SF3B1														
EIF1AX														
ATM														

footer: 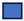 MUM (tumor specimen); 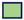 Primary UM (PBMCs*); 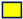 CHEK2; 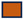 DNA damage repair gene; 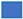 Gaq signaling pathway; 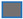 Other.

Case 1 to 3: Date from Thomas Jefferson University; Case 4 to 10 from Caris Life Science. *PBMCs: peripheral blood mononuclear cells.

### 3.3. Patients Without Metastatic UM (Primary Uveal Melanoma Patients Without Systemic Recurrence)

In addition to patients with UM metastasis, our database contained four primary uveal melanoma patients with *CHEK2* germline mutations.

#### 3.3.1. Case 11

A 66-year-old female presented with uveal melanoma in the right eye. She noticed reduced peripheral vision. She was subsequently diagnosed with choroidal melanoma. The tumor measured 16 mm in maximum diameter and 8.2 mm in thickness (stage IIB, T3aN0M0) at the age of 58, and she was treated with plaque radiation. Whole genome copy number analysis of DNA isolated from the primary uveal melanoma showed disomies of chromosomes 3 and 8, as well as chromosome 6p amplification. Her family history was significant for her paternal uncle and grandfather, who had uveal melanoma. In addition, her paternal aunt had breast cancer, and her uncle had prostate cancer. She had genetic counseling and germline testing completed, which reported pathogenic *CHEK2* mutations (c.1100delC:p.Thr367Mfs and c.7C > T:p.Arg3Trp). *BAP1*, *BRCA1*, and *BRCA2* germline mutations were negative. The patient is increasing the frequency of cancer screening. The patient has not had a local or distant recurrence of the disease for eight years after diagnosis of primary uveal melanoma.

#### 3.3.2. Case 12

A 57-year-old female presented with uveal melanoma in the left eye. She developed left eye photopsia. She was referred to a retina specialist and subsequently diagnosed with choroidal melanoma. The tumor measured 8 × 7 mm in base and 2.7 mm in thickness (stage I, T1aN0M0) at the age of 54, and the patient was treated with plaque radiation. The tumor was analyzed with whole genome copy number analysis of DNA isolated from the primary uveal melanoma, which showed mosaic monosomy 3 and disomies of chromosomes 6 and 8. Her personal and family history was remarkable for her brother, who was diagnosed with uveal melanoma and developed metastasis 10 years after his primary eye tumor diagnosis. He passed away roughly 15 months after developing systemic metastasis. Her mother had a meningioma. Her paternal grandmother has breast cancer, and her grandfather had lung cancer. Her maternal grandfather had an unknown cancer. She has one healthy sister. The patient also has a personal history of cervical cancer at the age of 43 and had a hysterectomy. She had genetic counseling and subsequently found a germline pathogenic *CHEK2* mutation (c.1100delC) and a *BAP1* (c.542_543delTT) mutation. *BRCA1* and *BRCA2* germline mutations were negative. She has a stable 1 cm mass in the right hepatic lobe favored to represent a flash-filling hemangioma or vascular malformation. The patient has not had a local or distant recurrence of the disease for four years after diagnosis of primary uveal melanoma.

#### 3.3.3. Case 13

A 46-year-old female presented with uveal melanoma in the right eye. She had a routine eye exam and identified an abnormality in the right eye. She was subsequently diagnosed with choroidal melanoma. The tumor measured 8 × 7 mm in base and 3.1 mm in thickness (stage I, T1aN0M0) at the age of 40, and the patient was treated with plaque radiation. The primary uveal tumor showed partial monosomy 3, as well as disomies of chromosomes 1, 6, and 8. Her personal and family history was remarkable for her mother, who was diagnosed with thyroid cancer. Her maternal grandmother had leukemia. Her son had sarcoma with a *CHEK2* mutation. She had genetic counseling and found a germline pathogenic *CHEK2* mutation (c.1215 C > A, p. N405K). There were no other cancer-related germline mutations, including *BAP1*, *BRCA1*, or *BRCA2*. The patient has not had a local or distant recurrence of the disease for seven years after diagnosis of primary uveal melanoma.

#### 3.3.4. Case 14

A 67-year-old female presented with uveal melanoma in the left eye. She had a routine eye exam and identified an amelanotic nevus in her left eye. She was subsequently diagnosed with choroidal melanoma. The tumor measured 10 × 8 mm in base and 2.7 mm in thickness (stage I, T1aN0M0) at the age of 67, and the patient was treated with plaque radiation. The tumor analysis showed disomies of chromosomes 3, 6, and 8. The patient has a personal history of two breast cancers. The first one was a stage IIA right breast carcinoma diagnosed at the age of 40. At the age of 54, she was found to have a stage 0 left breast carcinoma (LCIS versus DCIS). Her family history was significant for her mother and maternal aunt, as well as her brother’s daughter, who were all diagnosed with breast cancer. Her maternal grandfather had lymphoma. She had genetic counseling and found a germline *CHEK2* mutation (c.349A > G, p. R117G), which is considered a pathogenic missense mutation associated with an increased risk of developing certain cancers. Additionally, the patient has a germline *ATM* mutation (c.519G > T, p.R173S). Other cancer germline mutations, such as *BAP1*, *BRCA1*, or *BRCA2*, were negative. The patient has not had a local or distant recurrence of the disease for half a year after diagnosis of primary uveal melanoma.

Since we have not tested all primary uveal melanoma patients with germline *CHEK2* variations, we do not know the incidence of *CHEK2* variation in the general population of primary uveal melanoma patients.

## 4. Discussion

In this study, we report 10 patients among 740 MUM patients (1.4%) whose metastatic specimens exhibited variants in the *CHEK2* gene and four primary UM patients without systemic recurrence with *CHEK2* germline mutations. Although confirmatory germline testing with normal or non-malignant cells was not performed on the metastatic specimens, the *CHEK2* variants could be germline origin based on the high variable allele frequency (VAF). Three of these metastatic patients from our institution had at least one member in the family with clearly documented cancer. The sample number in this study is too small to make any broad conclusions; however, the median age of onset of primary UM with *CHEK2* variations in our database was 55 years old (n = 7, range 40–67), which is only slightly younger than the reported median age of onset of UM of 58 to 62 years [1,2,3]. To our knowledge, only two UM cases have previously been reported in the literature with a germline mutation in *CHEK2* [c.1100delC:p.Thr367Metfs and c.T470C:p.Ile157Thr] [19]. These patients were identified in a study of 154 UM patients with a high risk of hereditary cancer, using germline DNA analysis with WES. It is well known that a *BAP1* germline variant predisposes patients to UM and other cancers, including cutaneous melanoma, malignant mesothelioma, meningioma, and renal cell carcinoma [7,8,9]. *CHEK2* variants are also known to increase the risk of breast cancer, leukemia, and other cancers [26,27,28]. Variants in the *CHEK2* gene were first reported as truncating *CHEK2* mutations (c.1100delC) that confer a greater than twofold increase in the risk of breast cancer. Clinical studies evaluating the risk of cutaneous melanoma in *CHEK2* mutation carriers have been reported in the literature. The authors concluded that individuals with CHEK2*1100*delC* heterozygosity have a twofold risk of melanoma compared with non-carriers (OR 2.01 (1.03–3.91) in the Danish, 1.42 (0.46–4.31) in Germans, and 1.79 (1.02–3.17) in the Danish and Germans combined) [29]. In contrast, three common low-risk missense variants, I157T, S428F, and T476M, are not commonly associated with an increased risk of breast cancer [30,31]. These germline *CHEK2* variants occur in 0.6–1.1% of the general population [16,32]. Besides the well-known and most studied *CHEK2* mutations described above, other nonsense variants (e.g., Q20X, E85X) and missense mutations (H371Y and D438Y) have been associated with breast cancer [33,34]. Investigating primary UM cases to assess the incidence and risk of UM in individuals with *CHEK2* mutations may provide valuable insights.

The *CHEK2* variants with p.I157T in Case 1, E302K in Case 2, and p.S428F in Case 3 have been frequently observed as germline variants. I157T (470 T > C) in the FHA domain was reported in the normal Finnish population [35]. The American College of Medical Genetics and Genomics (ACMG) recently published *CHEK2* management guidelines highlighting that the low penetrance of I157T does not meet the 2-fold relative risk threshold for clinical significance [36]. The very rare *BRCA2* (c.475 + 1G > T) mutation was also found in Case 1. The role of *CHEK2* mutations in the presence of *BRCA2* mutations should be further investigated.

The *CHEK2* variant in Case 2 is located within a kinase domain (E302K). Myriad classifies it as likely pathogenic [37], but others classify it as a VUS [38,39]. There is still a need for further investigation of this variant to determine its level of pathogenicity. The risk of cancer associated with *CHEK2* missense variants is relatively low, with some notable exceptions. Data from the studies on the *CHEK2* (p.E302K) are insufficient to prove that it is one of these exceptions. It is of note that this patient also had a *MITF* variant. *MITF* (E318K) is also a low penetrance variant frequently observed in the germline of the general population. It is associated with a moderately increased risk of cutaneous melanoma [40]. In addition, Case 2 exhibited a deletion of the *PALB2* gene and a *BRCA1* (D695Y) mutation with a family history of cancer affecting four members, including one diagnosed with pancreatic cancer. The *CHEK2* S428F mutation in the kinase domain is a missense variant in Case 3. It is associated with lower rates of non-breast cancers [30,31]. Additionally, Case 3 has a *RAD51C* mutation that disrupts DNA repair.

It is important to note that the *CHEK2* variant data of MUM were analyzed from tumor tissue sections, and pathogenic somatic mutations in *GNA11* or *GNAQ*, along with *BAP1*, were detected alongside the *CHEK2* variants in the UM metastatic tissues. In general, the detectable time for metastasis in UM had been reported to peak differently in *BAP1* and *SF3B1* mutations. The earlier peaks appear to be associated with the *BAP1* mutation (usually between 1 and 3.5 years), while the later peak is most likely associated with the *SF3B1* mutation [41]. While Case 1, Case 2, and Case 3 all had somatic *BAP1* mutations in the tumor, the time it took to develop distant metastasis was very different. It took only 4 months after the primary diagnosis in Case 1, 1 year and 4 months in Case 3, and 14 years and 8 months in Case 2. A current limitation is the insufficient number of patients available to assess this interesting association with the *CHEK2* variant.

Although the role of *CHEK2* abnormalities in UM patients is not clear and needs to be further investigated, the presence of alternations in the ATM–CHK2 axis might justify the usage of previously untested medications in UM such as ataxia telangiectasia and Rad-3-related (ATR) kinase inhibitors and ATM kinase inhibitors, as *CHEK2* is part of the ATM–CHK2-p53 axis (Figure 1) [42,43]. Furthermore, the role of *CHEK2* abnormality in *BAP1*-mutated and *BRCA1/2*-mutated tumors should also be investigated to justify the usage of PARP inhibitors for UM tumors with these mutations [43,44,45]. Our findings may contribute to a better understanding of a potentially significant link between *CHEK2* mutations and UM patients to ultimately improve patients’ quality of health and provide implications for both early diagnosis and targeted treatments. To further elucidate the clinical implications of *CHEK2* variants, investigating a large cohort of primary UM cases would facilitate a comprehensive assessment of the incidence and clinical relevance of UM in affected individuals.

## 5. Conclusions

Investigations of a dysregulated CHK2 in UM could increase our understanding of its biological role and pave the way for personalized therapy for this disease. In this study, we do not have enough samples to conclude how these tumor-specific *CHKE2* variants may influence UM metastasis. Although rare, UM with abnormalities in the *CHEK2* mutation might benefit from inhibitors of DNA damage repair, warranting further investigation.

## Data Availability

The data analyzed in this study are subject to ethical restrictions: Raw data files cannot be made available due to patient confidentiality. Requests to access these datasets should be directed to the corresponding author.

## Supplementary Materials

The following supporting information can be downloaded at: https://www.mdpi.com/article/10.3390/jcm14082815/s1, Supplement Table S1: The summary of key DNA variant with site or deletion.

## Abbreviations

ACMG	American College of Medical Genetics and Genomics
ATM	ataxia-telangiectasia mutated kinase
ATR	ataxia telangiectasia and Rad-3 related
BAP1	BRCA1-associated protein-1
BCNU	carmustine
BRCA1/2	breast cancer gene 1/2
CAP	College of American Pathologists
CLIA	clinical laboratory improvement amendments
DDR	DNA damage response
EIF1AX	eukaryotic translation initiation factor 1A x-linked
FHA	forkhead-associated domain
GM-CSF	granulocyte-macrophage colony-stimulating factor
GNA11	guanine nucleotide-binding protein alpha 11
GNAQ	guanine nucleotide-binding protein, q polypeptide
IRB	Institutional Review Board
MBD4	methyl-CpG-binding domain protein 4
MSI	microsatellite instability
MUM	metastatic uveal melanoma
NGS	next-generation sequencing
PBMC	peripheral blood mononuclear cell
PKC	protein kinase C
PLCB4	phospholipase C beta 4
SCD	SQ/TQ cluster domain
SF3B1	Splicing Factor 3B Subunit 1
TMB	tumor mutational burden
TP53	tumor protein 53
UM	uveal melanoma
VAF	variable allele frequency
WES	whole exome sequencing
