# Supplementary material for: Short Report: The Variants in CHEK2 in Metastatic Uveal Melanoma
Source: J Clin Med. 2025 Apr 18;14(8):2815. doi: 10.3390/jcm14082815 (PMC12028195; doi:10.3390/jcm14082815)
Supplement: Supplementary file 1 [file jcm-14-02815-s001.zip › jcm-3568975-supplementary.pdf]

Supplement Table S1. The summary of key DNA variant with site or deletion

Case	Specimen Site	CHEK2	Other known DNA damage genes	Gaq	Other UM common genes	Detection Method
1*	Liver	I157T (47%) c.470T>C	BRCA2 (c.475+1G>T) (52%)	GNA11(Q209P) (14%)	BAP1 (p.S583fs) (13%)	NGS
2*	Liver	E302K (46%) c.904G>A	BRCA1 (p.D695Y) (44%), Deletion of PALB2	GNA11(Q209L) (43%)	BAP1 (c.68-13_82del28) (63%)	WES
3*	Liver	S428F (49%) (c.1283C.T)	RAD51C (p.Y216fs) (29%)	GNAQ (Q209P) (26%)	BAP1 (p.P510fs) (37%)	Hybrid WES
4	Lower lobe, lung	T383I (c.1148_1149delinsTT)	PALB2 (p.Y334C)	GNA11(R183C)	BAP1 (W5X)	NGS
5	Liver	D82_E86del		GNA11(Q209L)	BAP1 (Y546fs)	NGS
6	Liver	I157T(c.470T>C)		GNAQ (R183Q)	SF3B1 (R625C)	WES
7	Canthus, NOS	T367Mfs (c.1100delC)		PLCB4 (G517E)		WES
8	Liver	T367Mfs (c.1100delC)		GNA11(Q209P)	BAP1 (R56fs), SF3B1 (A263V)	WES
9	Middle lobe, lung	I157T (c.470T>C)		GNAQ (Q209P)	SF3B1 (R625H)	Hybrid WES
10	Liver	T367Mfs (c.1100delC)		GNAQ (Q209L)	EIF1AX (G9R)	Hybrid WES
11	#PBMC	T367Mfs (c.1100delC) Arg3Trp(c.7C>T)				
12	#PBMC	T367Mfs (c.1100delC)				
13	#PBMC	N405K (c.1215 C>A)				
14	#PBMC	R117G (c.349A>G)	ATM (p.R173S)			

* Case 1, 2 and 3: Data from Thomas Jefferson University
Case 4 to 10: Data from Caris Database
Case 11 to 14: Germline mutation analysis on peripheral blood mononuclear cells (PBMC) of primary uveal melanoma patients
#PBMC: Peripheral Blood Mononuclear Cells
NGS: 592 gene panel Next Generation Sequencing
WES: Whole Exome Sequencing
Hybrid WES: WES from the simul-capture approach
